# Effectiveness of *Streptomyces enissocaesilis* and chitosan on agronomic, biochemical, and quality traits of soybean under different irrigation intervals

**DOI:** 10.1186/s12870-026-08153-1

**Published:** 2026-02-11

**Authors:** Asmaa Hamoda, Mokhtar Dabbour, Sobhi F. Lamlom, Eman A. El-Akshar

**Affiliations:** 1 Department of Agronomy, Faculty of Agriculture, Benha University, P.O. Box 13736, Moshtohor, Qaluobia Egypt; 2 Department of Agricultural and Biosystems Engineering, Faculty of Agriculture, Benha University, P.O. Box 13736, Moshtohor, Qaluobia Egypt; 3 Plant Production Department, Faculty of Agriculture Saba Basha, Alexandria University, Alexandria, 21531 Egypt; 4 Department of Agricultural Microbiology, Faculty of Agriculture, Benha University, P.O. Box 13736, Moshtohor, Qaluobia Egypt

**Keywords:** Leaf area index, Oil content, Seed yield, Chlorophyll content, Proline content, Antioxidant enzyme activity

## Abstract

This study evaluated the growth, biochemical, yield-related, and chemical traits of soybean under different irrigation intervals (8, 13, and 18 days), seed inoculation with *Streptomyces enissocaesilis*, and foliar application of chitosan (0, 0.25, and 0.50 g.L^-1^) during two successive seasons in 2023 and 2024. A split-split plot design arranged in randomized complete blocks with three replications was employed. Analysis of variance indicated that irrigation interval was the most influential factor, exhibiting a highly significant effect (*p* < 0.001) on most assessed traits. Additionally, *Streptomyces* inoculation and chitosan application interacted synergistically, significantly improving all yield-related and chemical parameters compared to their individual effects. Under well-watered conditions (8-day interval), the combined application of seed inoculation with 0.50 g.L^-1^ chitosan resulted in the highest concentrations of chlorophyll a and b, more than doubling the levels observed in the stressed, untreated control. Conversely, the longest irrigation interval (18 days) combined with the same dual treatment triggered the strongest biochemical stress response, maximizing key markers including proline content, peroxidase, and polyphenol oxidase activities. Most notably, the highest 100-seed weight (23.33 g), pod number per plant (60.67), and pod weight per plant (38.00 g) were achieved under well-watered conditions with inoculation and 0.50 g.L^-1^ chitosan, resulting in the highest seed yield (3220.93 kg.ha^-1^). In contrast, water stress substantially compromised nutritional components, as evidenced by the significant reduction in oil and protein content, highlighting the critical role of inoculation and chitosan in preserving seed quality under such stress. These results were further validated by hierarchical clustering, principal component analysis, and radar plot visualization, confirming that the application of bio-stimulants (inoculation and chitosan) under well-watered conditions produced a distinct and favorable profile for soybean growth, biochemical, yield-related, and chemical traits. Therefore, the current findings provide valuable recommendations for the integrated application of *S. enissocaesilis* inoculation and chitosan under short-interval irrigation as a sustainable and effective strategy to maximize soybean productivity and quality.

## Introduction

With global food demand outpacing agricultural production, the search for reliable and nutritious crops is paramount. Soybean *(Glycine max (L.) Merr.)* is a key dual-purpose crop poised to meet this need, serving as a primary source of protein for humans, a high-quality animal feed, and a dependable source of essential dietary components. With a seed composition of 40–45% protein, 18–21% oil, and 26–30% carbohydrates [1], soybean is the leading oilseed crop worldwide, accounting for nearly 48% of total vegetable oil production [2, 3]. Its crude oil is composed of about 55% linoleic acid (18:2), 21% oleic acid (18:1), 12% palmitic acid (16:0), 9% linolenic acid (18:3), and 4% stearic acid (18:0). This favorable fatty acid profile, coupled with its rich nutritional profile, has driven unprecedented global demand. Consequently, the worldwide soybean harvest area more than doubled from 62.49 million ha in 1995 to 136.9 million ha in 2023. Despite its international importance, soybean cultivation in Egypt remains limited to 20,000 ha, with an average yield of 3 t.ha^-1^ [4]. This significant gap between global adoption and local production underscores the critical need to identify and implement effective agronomic solutions to expand cultivation area and increase production in Egypt under semi-arid and arid climate conditions.

Abiotic stresses substantially limit crop productivity, with climate-driven extremes such as drought, salinity, and heat posing growing risks to food security [5]. Among these, water limitation is a major and pervasive challenge, especially in ecosystems with already low baseline moisture availability. Future climate projections indicate that arid and semi-arid areas will experience the greatest increases in water scarcity, further heightening crop vulnerability [6]. Therefore, efforts to expand soybean cultivation are severely challenged by the pressures of a changing climate. Compounding this issue, climate change is causing prolonged periods of high temperatures during arid summer conditions, which expose the soybean crop to extreme heat and prolonged water stress [7]. This combined stress induces physiological damage, primarily by disrupting cell division in meristematic regions, thereby adversely affecting both plant growth and overall productivity [8]. Under such water-limited conditions, soybean yield can decrease by about 40% [9]. Water stress impairs plant growth and development through mechanisms such as triggering oxidative damage, altering key enzyme activities, and compromising cell membrane integrity [10]. This physiological damage directly translates into yield loss, which is significantly linked to the irrigation-deficit-induced abortion of flowers and pods [11]. The timing of the stress is also critical, as it determines which developmental stage, and consequently which yield components, are most severely compromised. For instance, Desclaux et al. [12] found that stress during the early seed-filling stage reduced the number of seeds per pod, while the late-stage stress decreased seed weight. Under water-limited conditions, plants often modify their root system to optimize water acquisition. This shift favors the production of thinner branch roots over the elongation of axile roots, likely because generating more root length per unit carbon is more efficient under stress [13, 14]. Given the severe threat posed by increasing water stress, developing strategies to mitigate its impact is crucial. In this context, research has increasingly focused on sustainable agriculture, with one promising approach being the use of plant growth-promoting rhizobacteria (PGPR) to enhance crop stress tolerance [15].

PGPR are beneficial soil microbes that colonize the rhizosphere, where root exudates provide the nutrients to sustain their metabolism [16]. These bacteria directly stimulate both root development (e.g., root length, root hair density, and overall system growth) and plant growth by facilitating resource acquisition through nitrogen fixation and increased mineral uptake, as well as by modulating plant hormone levels [17, 18]. Furthermore, the PGPR’s ability to form biofilms in these nutrient-rich niches amplifies their beneficial effects, leading to improved nutrient and water uptake and enhanced plant stress resilience [19]. Several microorganisms, including strains from the genera *Bacillus*, *Bradyrhizobium*, *Trichoderma*, *Pseudomonas*, and *Streptomyces*, have recently demonstrated promising results in enhancing drought tolerance [20, 21]. *Streptomyces* species, key decomposers of organic matter, produce beneficial secondary metabolites such as cellulase, phytase, and phosphatase, which contribute to improved nutrient cycling and plant health. They also augment plant tolerance to water stress by modulating the expression of antioxidant enzymes and drought-resistant genes [22, 23]. Supporting this, Shan et al. [24] observed that *Streptomyces* inoculation significantly enhanced wheat plant growth under water stress. Most importantly, *S. enissocaesilis* possesses distinct physiological advantages for mitigating drought stress. Unlike many rhizobacteria, this strain exhibits a robust synergistic profile, combining strong root colonization with the prolific secretion of osmolytes, antioxidant-inducing metabolites, and stress-responsive phytohormones [25, 26]. This multi-faceted action collectively enhances plant resilience to abiotic stress.

Furthermore, the biodegradable compound chitosan offers another effective strategy for enhancing drought resilience. This non-toxic polymer is a suitable alternative to synthetic agrochemicals due to its excellent film-forming and water retention properties [27]. It represents an up-and-coming tool for sustainable agricultural application and improved crop production [28]. Chitosan bolsters plant defense systems against various abiotic stresses. Under water deficit conditions, specifically, it mitigates water deficit effects by boosting antioxidative enzyme activity, improving water-use efficiency through increased root growth, and promoting photosynthetic activity [29]. Additionally, Almeida et al. [30] and Lavinskya et al. [31] found that chitosan significantly promoted root development and crop growth under water deficit by improving the availability and absorption of water and nutrients, which is linked to cellular osmotic adjustment. Research on soybeans demonstrates this protective effect. For example, Esmaeeli et al. [11] noted that deficit irrigation substantially decreased soybean seed yield, oil content, and protein content by 60.0%, 12.5%, and 23.3%, respectively, compared to the well-watered control. However, chitosan application mitigated these losses, increasing the same parameters by up to 6.9, 8.1, and 6.7%, respectively. Similarly, Methela et al. [32] concluded that applying chitosan to water-stressed soybeans increased root length, area, and volume, plant height, and total biomass. They also observed an increase in catalase and ascorbate peroxidase activity following chitosan treatment (50 µM). As a result, key indicators of oxidative stress, including electrolyte leakage, malondialdehyde, and hydrogen peroxide, were significantly decreased under such conditions.

The dual challenges of ensuring food security and mitigating water scarcity in semi-arid regions such as Egypt necessitate innovative approaches to optimize agricultural production. Although beneficial microbes and chitosan individually show promise for enhancing drought tolerance, their interactive effects under realistic, varying water stress conditions remain underexplored. Specifically, it is unclear whether the integration of *S. enissocaesilis* with chitosan can produce synergistic benefits for key agronomic and physiological traits, or how such an interaction is modulated by different levels of irrigation stress to sustain soybean productivity. To address this critical gap, this study systematically examined the combined effects of *S. enissocaesilis* inoculation and foliar chitosan (0, 0.25, and 0.50 g.L^− 1^) across different irrigation intervals (8, 13, and 18 days) on the growth, biochemical, yield-related, and chemical parameters of soybean. The interrelationships among the studied soybean traits and the applied treatments were also elucidated through hierarchical clustering, principal component analysis (PCA), and radar plot visualization. The outcomes of this work provide a sustainable and practical strategy for farmers and breeders to stabilize soybean productivity under water-limited conditions.

## Materials and methods

### Study location, soil characteristics, and Climatic conditions

**Fig. 1:**
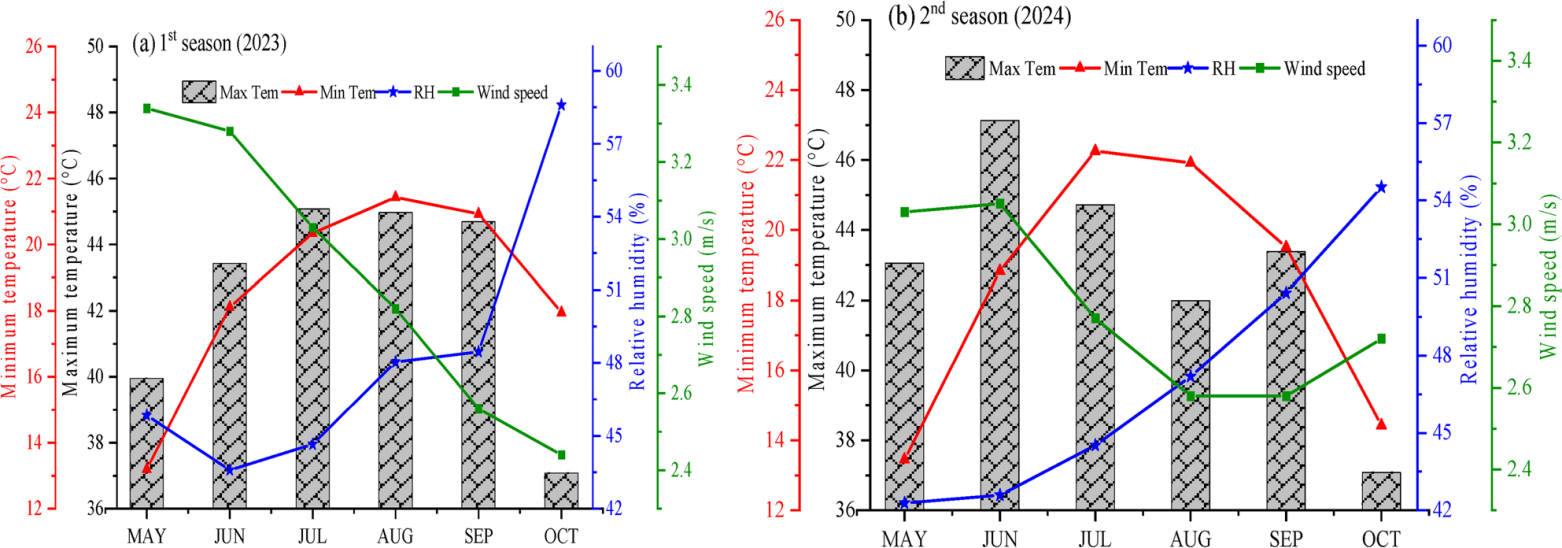
Monthly variations in climatic conditions at the experimental site during the 2023 and 2024 growing seasons, showing maximum and minimum temperature, relative humidity, and wind speed. Data were obtained from the NASA POWER database (https://power.larc.nasa.gov/data-access-viewer/)

The study was conducted over two consecutive growing seasons (2023 and 2024) at the Research and Experimental Station, Faculty of Agriculture, Benha University, Egypt (30.45° N, 31.10° E). Prior to the experiment, ten soil samples were randomly collected from the experimental field using a spiral auger at 0–30 cm and 30–60 cm depths. For each depth, the samples were composited, homogenized, air-dried, ground, and sieved through a 2-mm mesh. Subsequently, three representative subsamples were taken from each composited sample for analysis. The physicochemical properties of soil samples were analyzed as previously described [33, 34]. Table 1 presents the physicochemical properties of the soil for both growing seasons. The soil at the experimental site was a loamy clay with high organic matter content (13.40–21.40 g kg^−^¹). Key physicochemical properties were consistent across both growing seasons. The soil pH was slightly alkaline, ranging from 7.60 to 8.00. The particle size analysis revealed a texture dominated by clay (49–51.33%), followed by silt (24.4–25.70%), fine sand (17.97–19.17%), and coarse sand (5.80–6.30%). The soil exhibited low electrical conductivity (EC < 0.16 dSm^−^¹), indicating minimal salinity. Analysis of the soil solution identified calcium (Ca²⁺: 1.18–2.06 mmol_c_ L^−^¹) as the dominant cation, followed by magnesium (Mg²⁺: 1.09–2.26 mmol_c_ L^−^¹) and potassium (K⁺: 0.82–1.45 mmol_c_ L^−^¹). The primary anions were bicarbonate (HCO₃⁻:1.50–2.09 mmol_c_ L^−^¹) and chloride (Cl⁻: 1.30–2.30 mmol_c_ L^− 1^). Figure 1 shows the climatic data recorded during the soybean growth periods. From May to September, the mean minimum temperature was 18.66 °C in 2023 and 18.40 °C in 2024. The mean maximum temperature for the same period was 42.53 °C and 42.89 °C, respectively, while the average relative humidity was 48.20% and 46.92%, respectively. Wind speed was similar in both growing seasons, averaging 2.91 m.s^− 1^ in 2023 and 2.79 m.s^− 1^ in 2024.

**Table 1:** Physicochemical characteristics of the experimental site during the 2023 and 2024 seasons

Properties	2023		2024	
	0–30 cm	30–60 cm	0–30 cm	30–60 cm
EC (dSm^-1^)	0.16	0.79	0.25	0.76
pH (1:2.5 w/v)	7.60	7.84	7.79	7.91
Organic matter (g.kg^-1^)	15.50	21.40	13.40	18.20
Soluble cations (mmol_c_ L^-1^)				
Ca^++^	2.06	1.18	2.00	1.23
Mg^++^	2.20	1.09	2.26	1.14
K^+^	0.85	1.40	0.82	1.45
Soluble anions (mmol_c_ L^-1^)				
Cl^-^	1.21	2.30	1.30	2.27
CO_3_^- -^	0.00	0.00	0.00	0.00
HCO_3_^-^	2.00	1.64	2.09	1.50
Particle size distribution (%)				
Fine sand (%)	17.97	19.17	18.65	19.10
Coarse sand (%)	5.80	6.30	5.85	6.20
Silt (%)	24.90	25.23	24.40	25.70
Clay (%)	51.33	49.30	51.10	49.00
Textural class *	Clay	Clay	Clay	Clay

### Experimental design and treatments

**Fig. 2:**
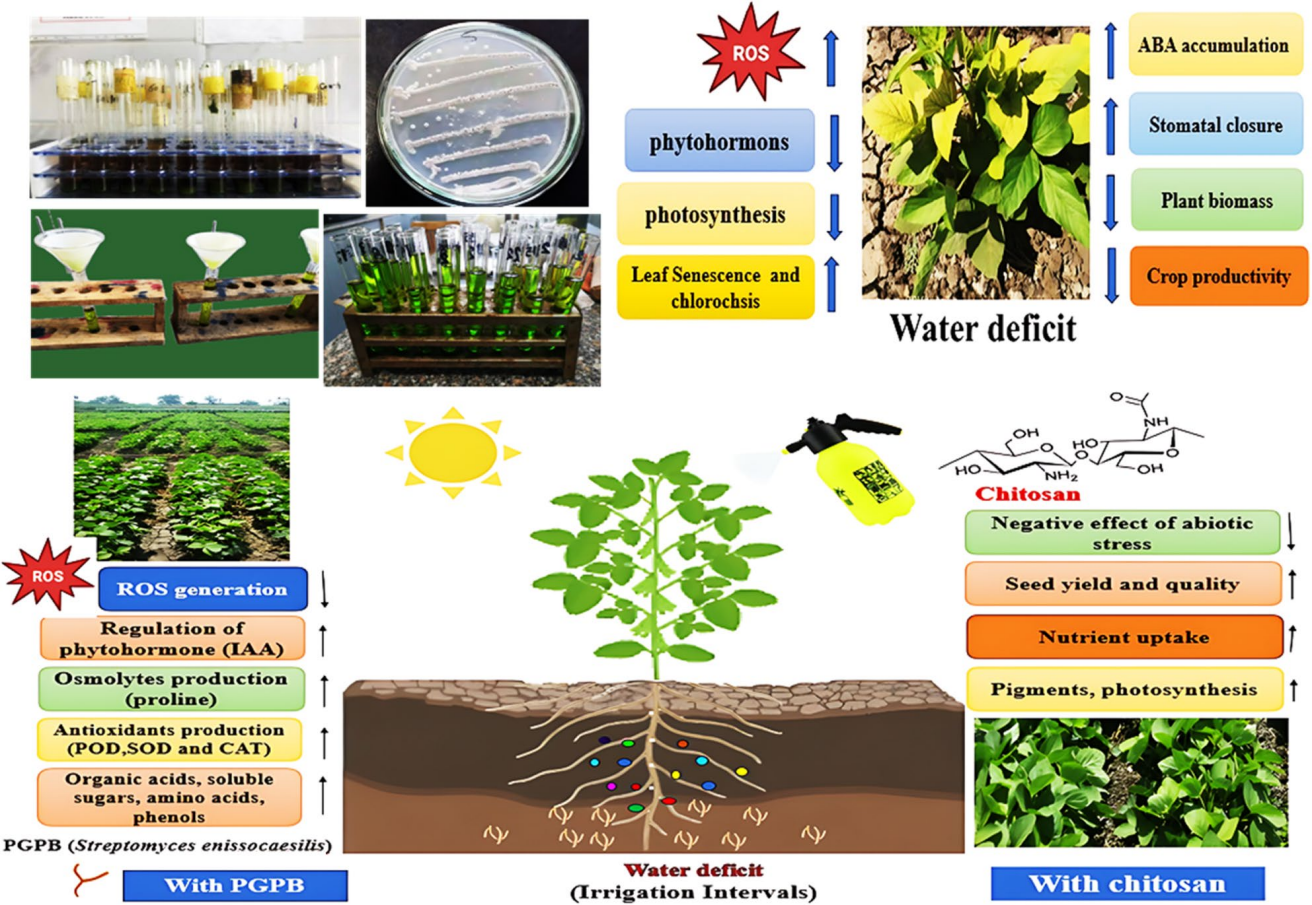
Schematic diagram illustrating the experimental design for evaluating the combined effects of water deficit, *Streptomyces* inoculation, and chitosan application on soybean

The experiment was arranged in a split-split-plot design, structured as a randomized complete block design with three replications. This design, with 3 irrigation intervals × 2 inoculation treatments × 3 chitosan concentrations, resulted in a total of 54 experimental sub-sub-plots. Irrigation intervals were assigned to the main plots, with intervals of 8, 13, and 18 days chosen to simulate a gradient of water stress. The 8-day interval served as a well-watered control, while the longer intervals (13 and 18 days) were used to induce moderate and severe moisture deficits, simulating conditions of water stress, particularly during peak summer temperatures. The sub-plots were allocated to seed inoculation treatments: (1) control (non-inoculated) and (2) inoculation with *S. enissocaesilis*. Three chitosan concentrations [0 (distilled water as control), 0.25, and 0.50 g.L^− 1^] were applied to the sub-sub-plots as a foliar spray. Chitosan concentrations were selected based on ranges established in a previous soybean study [11], which demonstrated their efficacy in eliciting growth and stress responses without phytotoxicity. Foliar application was carried out at 45 days after planting (DAP) in the morning using a hand sprayer at a rate of 952 L.ha^− 1^ for each plot. Each experimental plot consisted of five 3-meter-long ridges spaced 60 cm apart. The two outer rows (1st and 5th ) in each plot served as borders, while the central three rows were designated as the net plot for data collection. A schematic diagram illustrating the experimental design for evaluating the combined effects of irrigation intervals, *Streptomyces* inoculation, and chitosan application on soybean is presented in Fig. 2.

### Preparation and application of *Streptomyces* inoculum

#### Water stress tolerance assay

The *Streptomyces enissocaesilis* strain OM182843, previously isolated and identified in our work [26], was used in this study. The plant growth-promoting traits of the strain, including the production of gibberellic acid, indole-3-acetic acid, exopolysaccharides, and siderophores, were previously characterized [25]. The ability of *S. enissocaesilis* to grow under water stress was assessed on International *Streptomyces* Project medium 4 (ISP-4). The medium contained (g L^−^¹): soluble starch, 10.0; MgSO₄, 1.0; K₂HPO₄, 1.0; (NH₄) _₂_SO₄, 2.0; CaCO₃, 2.0; NaCl, 1.0; agar, 20.0; and trace salts (including FeSO₄·7 H₂O, 0.001; MnCl₂, 0.001; ZnSO₄·7 H₂O, 0.001). The pH was adjusted to 7.5 ± 0.2. Water stress was simulated by supplementing the medium with polyethylene glycol (PEG 6000) at concentrations of 10%, 15%, 20%, 25%, and 30% (w/v). A 7-day-old culture of *S. enissocaesilis* was streaked onto PEG-amended plates and incubated statically at 37 °C for 7 days [35].

#### Inoculum Preparation and seed treatment

For inoculum production, 1.0 L of ISP-4 broth in a 2.0 L Erlenmeyer flask was inoculated with *S. enissocaesilis* and incubated at 35 ± 0.2 °C for 7 days on a rotary shaker at 150 rpm. After incubation, the culture was homogenized by vigorous shaking with sterile glass beads to disperse spores evenly. Spore concentration was determined using a hemocytometer under a light microscope at 800× magnification and adjusted to 9 × 10⁹ conidia mL^−^¹ with sterile distilled water to serve as the inoculant carrier. Soybean seeds were surface-sterilized with 70% ethanol for 2 min, rinsed thoroughly with sterile distilled water, and subsequently immersed in the adjusted *S. enissocaesilis* spore suspension for 60 min before planting. This procedure constituted a single seed treatment applied at sowing.

#### Agronomic practices

The soybean cultivar Giza 111 was used in this study. Seeds were procured from the Field Crops Research Institute of the Agricultural Research Center (ARC), Ministry of Agriculture and Land Reclamation, Egypt. The seeds were sown at a rate of 72 kg.ha^− 1^ and placed at a soil depth of 3–5 cm. They were sown on May 22 and May 25 in the first and second season, respectively, and harvested in October of both seasons. Fertilization and crop management were conducted as follows: Before planting, a basal application of phosphorus was made using commercial single superphosphate fertilizer (15.5% P₂O₅) at 357 kg.ha^− 1^ during land preparation. Afterward, nitrogen was applied as urea fertilizer (46.5% N) at 50 kg.ha^− 1^. At three weeks post-planting, thinning was performed to maintain two plants per hill. All other agronomic practices followed the standard soybean cultivation recommendations issued by the Ministry of Agriculture, Egypt.

### Data collection and measurements

#### Growth traits

Growth traits of soybean were examined as previously detailed by Hamoda et al. [36]. At 75 DAP, five representative plants were randomly sampled from each plot across the three replications. The following growth traits were measured: plant height (cm), measured from the soil surface to the plant apex; and the number of branches per plant, defined as the count of primary branches on the main stem. The total leaf area per plant was measured using a CI-203 Handheld Laser Leaf Area Meter (CID Bio-Science, USA), and the leaf area index (LAI) was then calculated as the ratio of the measured leaf area (m^2^) to the ground area (m^2^) occupied by the plant. The number of pods per plant was also recorded. For dry weight measurements, the fresh leaves, stems, and pods were placed in paper bags and dried in an oven at 70 °C for 72 h. Following drying, the dry weight of each component was directly measured using a digital balance (YHC weighing excellence, Wonderscales, China).

#### Chlorophyll a and b content

Chlorophyll a and b were quantified following the protocol of Arnon [37] with slight modifications. For each treatment, leaf samples were collected from three independent replicate plots. A 0.5 g of composite fresh leaf tissue from each plot was homogenized in 20 mL of 80% acetone. The absorbance of the extract was spectrophotometrically measured at 663 and 645 nm. Chlorophyll a and b concentrations were calculated as follows:

$$\:Chlorophyll\:a\:\left(mg.{g}_{Fw}^{-1}\right)=\left[\left(12.7\times\:{A}_{663}\right)-\left(2.69\times\:{A}_{645}\right)\right]\times\:\frac{V}{w\times\:1000}$$

$$\:Chlorophyll\:b\:\left(mg.{g}_{Fw}^{-1}\right)=\left[\left(22.9\times\:{A}_{645}\right)-\left(4.7\times\:{A}_{663}\right)\right]\times\:\frac{V}{w\times\:1000}$$

where V represents the total extract volume (mL); w represents the fresh weight of the sample (g); and A_663_ and A_645_ represent the absorbance at 663 and 645 nm, respectively.

### Relative water content (RWC)

The RWC was examined as previously outlined [38]. Leaf discs were excised from the center of the leaves and immediately weighed to obtain the fresh weight (FW). The discs were then soaked in distilled water for 4 h and weighed again to determine the turgid weight (TW). Subsequently, the samples were oven-dried at 70 °C for 72 h until a constant dry weight (DW) was achieved. The RWC (%) was computed as follows:

$$\:RWC\:\left(\%\right)=\frac{FW-DW}{TW-DW}\times\:100$$

### Proline content

The protocol of Bates et al. [39] was followed to quantify the proline content in soybean leaves. Fresh leaf samples (0.5 g) were ground using a mortar and pestle, suspended in sulphosalicylic acid (10 mL, 3% *w/v*), and then filtered through filter paper. A 2 mL aliquot of the filtrate was mixed with 2 mL of glacial acetic acid and 2 mL of acid-ninhydrin. The resultant was incubated in a water bath at 100 °C for 1 h, then rapidly cooled in an ice bath. Thereafter, 4 mL of toluene was added, and the mixture was vortexed vigorously for 20 s. After the toluene and aqueous phases separated, the absorbance of the toluene (upper) layer was measured at 520 nm. The proline concentration was determined using a standard curve and calculated on a fresh weight basis as follows:

$$\:Proline\:content\:(mg.{g}_{Fw}^{-1})=\frac{C\:\times\:v}{\mathrm{w}}$$

where C is the proline concentration (mg.mL^− 1^); V is the total volume of the toluene extract (mL); and W is the fresh weight of the leaf sample (g).

### Antioxidant enzyme activity

Enzymes were extracted from 0.5 g of fresh leaf tissue collected from 8-week-old plants. The tissue was homogenized in 5.0 mL of 0.2 M Tris-HCl buffer (pH 7.8) containing 14 mM β-mercaptoethanol at a 1:10 (w/v) ratio. The homogenate was centrifuged at 10,000 𝗑 g for 20 min at 4 °C. The resulting supernatant was collected, aliquoted, and stored at -20 °C. All enzymatic assays were performed within 48 h of extraction to ensure stability and maintain activity.

### Peroxidase activity

Peroxidase activity was assayed according to the method of Allam and Hollis [40]. A 0.3 mL aliquot of enzyme extract was transferred to a 5 mL test tube. Then, 0.5 mL potassium phosphate buffer (100 mM, pH 7.0), 0.3 mL pyrogallol (50 mM), and 0.1 mL H_2_O_2_ (1.0%) were added. The final volume was adjusted to 3.0 mL with distilled water. After incubation at 25 °C for 15 min, the reaction was terminated by adding 0.5 mL of H_2_SO_4_ (5.0%). Absorbance was measured immediately at 425 nm. Peroxidase activity was calculated as the change in absorbance per gram fresh weight per 15 min (∆A₄₂₅g^−^¹ FW 15 min^−^¹).

### Polyphenol oxidase activity

Polyphenol oxidase (POA) activity was evaluated using the method of Matta and Dimond [41] with some modifications. Briefly, 0.2 mL of enzyme extract, 1.0 mL of sodium phosphate buffer (0.2 M, pH 7.0), and 1.0 mL of catechol (1.0 mM) were mixed in a test tube. The final volume was brought to 6.0 mL with distilled water. The solution was allowed to react for 30 min at 30 °C, and the absorbance was analyzed spectrophotometrically at 420 nm POA activity was expressed as the change in absorbance per gram fresh weight per 30 min (∆A₄₂₀ g^−^¹ FW 30 min^−^¹).

### Yield components

At physiological maturity (120 DAP), soybean plants were harvested. To evaluate yield components, five plants were randomly sampled from the three central ridges of each experimental plot across all three replications. The number of pods per plant, pod weight (g/plant), and 100-seed weight (g) were measured. The seed yield was subsequently calculated on an area basis and is expressed as kg/ha.

### Chemical analysis

#### Protein content

The nitrogen content of soybean seeds was determined using the Kjeldahl method, as previously described by Hamoda and Dabbour [42]. A 0.2 g sample of seed powder was digested with a mixture of sulfuric and perchloric acids. The digestion mixture was heated gradually to ~ 370 °C until a clear, colorless solution was obtained. The resulting digest was diluted to a final volume of 50 mL with distilled water. The diluted digest (10 mL) was then distilled with 20 mL of sodium hydroxide, and the liberated ammonia was trapped in 25 mL of boric acid containing a mixed indicator. The trapped ammonia was subsequently titrated with 0.1 N hydrochloric acid. The crude protein content was calculated by multiplying the nitrogen content by a conversion factor of 5.71.

### Oil content

The oil content was determined by solvent extraction using a Soxhlet apparatus, as previously described [43]. A 5 g sample of seed powder was placed in a cellulose thimble and loaded into the extractor. Petroleum ether (150 mL, boiling point 40–60 °C) was added to a dry, pre-weighed round-bottom flask, which was then attached to the apparatus. The system was heated using a mantle set to 65 °C to maintain a condensation rate of 2–3 drops per second for 3 h, ensuring approximately 18–22 complete extraction cycles. Upon completion, the solvent was evaporated, and the crude soybean oil was dried in an oven at 105 °C to constant weight to remove residual solvent. The flask was then cooled in a desiccator and reweighed. The oil content was calculated as follows:

$$\:Oil\:content\:\left(\%\right)=\frac{{m}_{1}-{m}_{2}}{{m}_{1}}\times\:100$$

where m_1_ is the mass of the sample before extraction (g) and m_2_ is the mass of the sample after extraction (g).

### Data analysis

Data from the two growing seasons were subjected to a full factorial three-way ANOVA using MSTAT-V21 software, analyzing the main effects of Irrigation, Microbe, and Chitosan, and all two- and three-way interactions. The assumption of homogeneity of variance was verified with Bartlett’s test prior to ANOVA. Significant differences between the means were determined via Tukey’s comparison test at *p* < 0.05, and the results are presented as means ± standard deviation. Hierarchical clustering, principal component analysis (PCA), and radar plots were performed to elucidate the interrelationships among the measured parameters and the different treatments. All figures were generated using Origin Pro 2023b software (Origin Lab Corporation, MA, USA).

## Results and discussion

### Effects of irrigation intervals, *Streptomyces* inoculation, and chitosan on growth traits

The ANOVA results (Table 2) showed that irrigation, *Streptomyces* inoculation, and chitosan application had highly significant (*p* < 0.001) main effects on most growth traits, including plant height, pod number per plant, and pod dry weight. However, the dry weight of leaves was not significantly impacted (*p* > 0.05) by inoculation, nor was branch number affected by chitosan. Significant two-way interactions further modulated responses. The irrigation × inoculation interaction improved all traits except leaf and stem dry weight. Interactions involving chitosan (either with irrigation or inoculation) strongly increased plant height, pod number, and pod dry weight, though leaf area index was unaffected. Most importantly, the three-way interaction (irrigation × inoculation × chitosan) was statistically significant, demonstrating a synergistic effect on key growth traits: plant height, pod number, and pod dry weight (*p* < 0.001), with a lesser effect on leaf area index (*p* < 0.05). Branch number, however, remained unresponsive to this interaction.

**Table 2:** Analysis of variance (ANOVA) for the effects of irrigation intervals, *Streptomyces* inoculation, and Chitosan application on growth traits of soybean

Source of variance	d.f	Plant height	Number of branches per plant	Leaf area index	Dry weight of Leaves	Dry weight of stems	Dry weight of pods per plant	Number of pods per plant
Blocks	2	1.64 ^ns^	0.09 ^ns^	0.76 ^ns^	0.18 ^ns^	1.04 ^ns^	2.29 ^*^	0.18 ^ns^
I	2	637.87 ^***^	13.89 ^***^	12.43 ^**^	175.71 ^***^	212.03 ^***^	688.85 ^***^	550.51 ^***^
Error (a)	4	3.52	0.13	0.29	1.36	0.75	0.21	0.77
B	1	261.31 ^***^	2.44 ^**^	2.83 ^**^	1.24 ^ns^	19.29 ^**^	223.26 ^***^	210.43 ^***^
I × B	2	13.47 ^*^	0.53^*^	1.21 ^*^	2.01 ^ns^	2.11 ^ns^	20.92 ^**^	51.17 ^***^
Error (b)	6	1.57	0.09	0.16	0.69	0.75	0.94	0.27
C	2	325.77 ^***^	0.14 ^ns^	2.12 ^*^	39.27 ^***^	54.62 ^***^	91.54 ^***^	427.70 ^***^
I × C	4	48.51 ^***^	0.26^**^	0.36 ^ns^	51.77 ^***^	24.30 ^***^	43.33 ^***^	127.15 ^***^
B × C	2	0.46 ^***^	0.83 ^***^	1.25 ^ns^	3.24 ^*^	2.90 ^*^	7.57 ^***^	113.62 ^***^
I × B × C	4	8.36 ^***^	0.06 ^ns^	1.40 ^*^	17.57 ^***^	17.03 ^***^	4.45 ^***^	14.44 ^***^
Error (c)	24	1.25	0.06	0.41	0.60	0.69	0.63	0.69

I = Irrigation intervals, B = *Streptomyces* inoculation, C = Chitosan application

*, **, *** = Significant at *p* < 0.05, *p* < 0.01, and *p* < 0.001, respectively; ns = non-significant

**Fig. 3:**
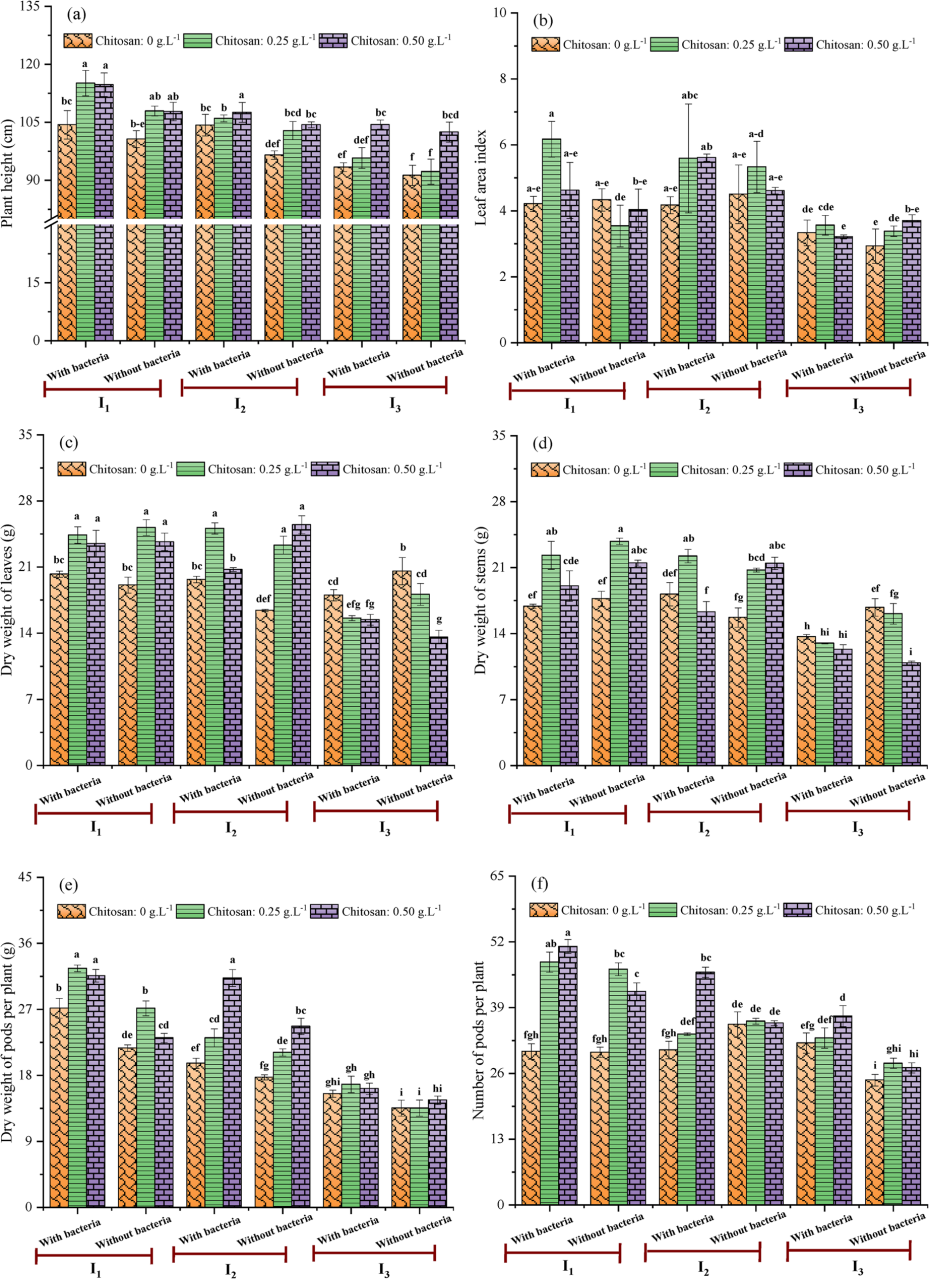
Combined effects of irrigation intervals, *Streptomyces* inoculation, and chitosan on plant height (**a**), leaf area index (**b**), dry weight of leaves (**c**), dry weight of stem (**d**), dry weight of pods (**e**), and number of pods per plant (f) of soybean. I_1_ = Irrigation every 8 days, I_2_ = Irrigation every 13 days, I_3_ = Irrigation every 18 days. Different letters on the bars denote statistically significant differences according to Tukey’s test at *p* < 0.05

**Fig. 4:**
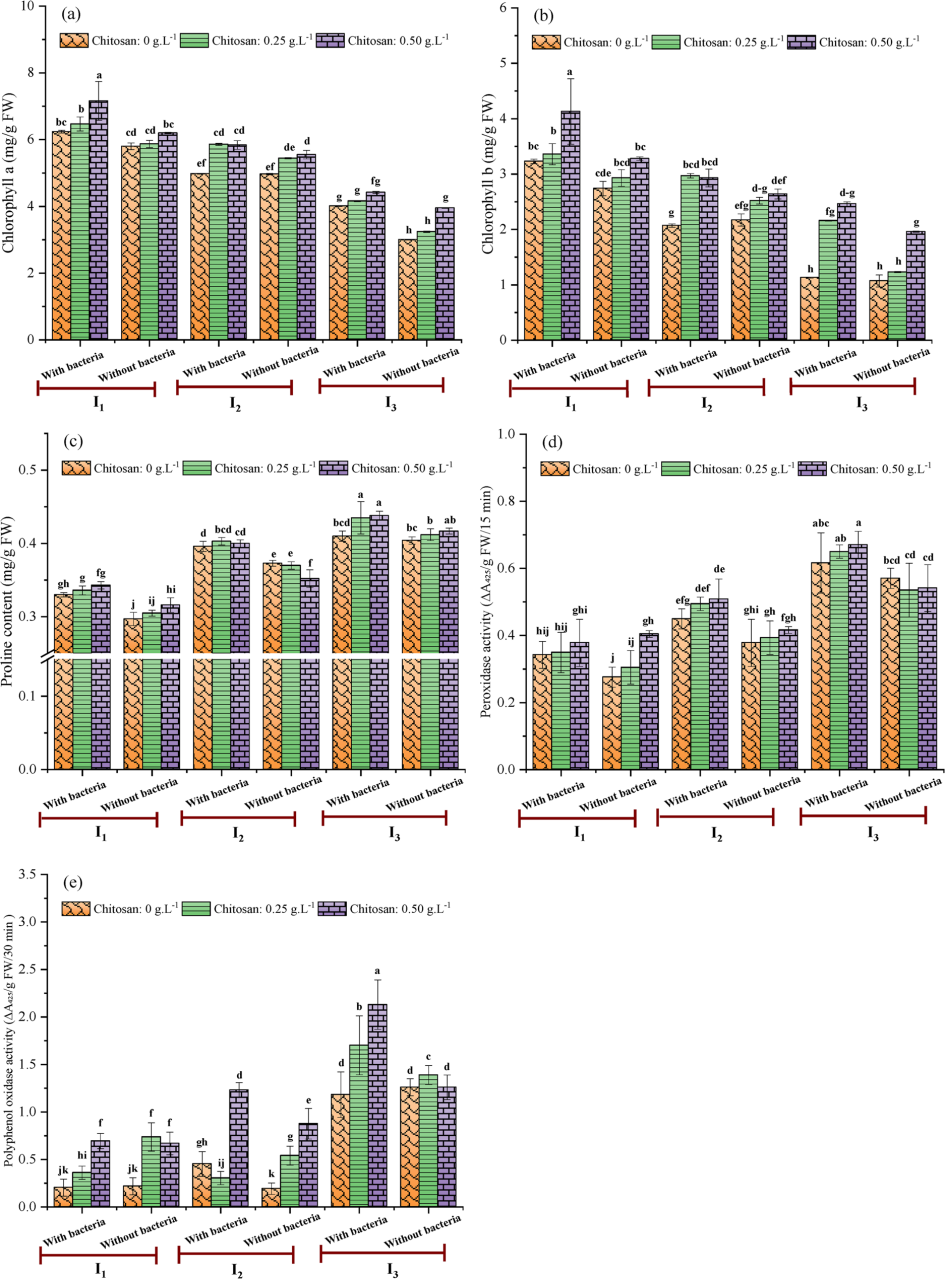
Combined effects of irrigation intervals, *Streptomyces* inoculation, and chitosan on chlorophyll a (**a**), chlorophyll b (**b**), proline content (**c**), peroxidase activity (**d**), and polyphenol oxidase activity (**e**) of soybean. I_1_ = Irrigation every 8 days, I_2_ = Irrigation every 13 days, I_3_ = Irrigation every 18 days. Different letters on the bars denote statistically significant differences according to Tukey’s test at *p* < 0.05

Figure 3a–f presents the synergistic effects of irrigation intervals, seeds inoculation with *Streptomyces*, and chitosan application on soybean growth parameters. The results revealed that the tallest plants (115.14 ± 3.30 cm) were found under the 8-day irrigation interval (I_1_) when treated with *Streptomyces* inoculation and chitosan at 0.25 g.L^− 1^ (Fig. 3a). Interestingly, plants in the I_1_ and I_2_ treatments that were inoculated with *Streptomyces* and sprayed with chitosan (at 0.50 g.L^− 1^) showed no significant difference in height. This stands in sharp contrast to the significantly shorter plants (91.26 ± 2.62 cm) in the stressed control (I_3_, 18-day interval), which lacked both bacterial inoculation and chitosan application. The observed reduction in plant height under stress is mainly attributed to limited water availability, leading to a decrease in turgor pressure, which is the primary driver of cell expansion, thereby impeding stem elongation and overall plant growth, a phenomenon previously reported in soybean by Zhao et al. [44]. The superior performance of the combined *Streptomyces*-chitosan treatment likely stems from their synergistic ability to mitigate water stress. Both treatments stimulate plant growth under drought by enhancing water and nutrient uptake, strengthening antioxidant systems to scavenge reactive oxygen species (ROS), and promoting the production of growth-regulating phytohormones [45, 46]. Specifically, *Streptomyces*, as a PGPR, further facilitates resource acquisition through nitrogen fixation and phosphate solubilization. For instance, under water stress, PGPR inoculation has been shown to reduce shoot growth inhibition from 41% to 18% by improving nutrient availability and resilience [47]. In this study, foliar chitosan similarly alleviated stress, improving soybean plant height and other growth metrics. These outcomes were consistent with findings that chitosan application boosted root development, biomass, and plant height in water-stressed soybeans [32]. The leaf area index (LAI), a key morphological indicator of water stress, was significantly influenced by the treatments. The most favorable interaction was observed under the I_1_ irrigation (8 days) combined with bacterial inoculation and 0.25 g.L^− 1^ chitosan, which produced an LAI of 6.17 ± 0.54 (Fig. 3b). This value was more than double that of the most stressed treatment (I_3_, without bacteria or chitosan), which had an LAI of only 2.93 ± 0.52. This dramatic difference demonstrates the pronounced efficacy of the combined bio-stimulants in mitigating water stress. These observations were likely mediated by the treatment-induced modulation of the auxin/cytokinin balance, a key regulator of cell division and expansion. Both chitosan and *Streptomyces* are known to influence this hormonal equilibrium. Chitosan directly affects auxin and cytokinin signaling pathways, promoting leaf development. Concurrently, specific *Streptomyces* strains, recognized as effective PGPR, have been shown to significantly enhance soybean leaf area [48], potentially through similar hormonal adjustments. This synergistic hormonal modulation by the combined treatment provides a mechanistic explanation for the superior leaf area enhancement observed in our study. For dry weight of leaves, the highest value (25.48 ± 0.95 g) was observed in non-inoculated plants irrigated every 13 days (I_2_) and treated with 0.50 g.L^− 1^ chitosan (Fig. 3c). This was statistically similar to the value from well-watered (I_1_), non-inoculated plants sprayed with 0.25 g.L^− 1^ chitosan (25.18 ± 0.84 g). Both treatments yielded significantly higher leaf dry weight than all plants under the long irrigation interval (I_3_), regardless of inoculation or chitosan concentration, which ranged from 13.57 ± 0.72 to 20.56 ± 1.44. Furthermore, well-watered (I_1_), non-inoculated plants sprayed with 0.25 g.L^− 1^ chitosan exhibited the heaviest dry weight of stems (23.79 ± 0.34 g) (Fig. 3d). This was substantially greater than the 10.90 ± 0.20 g recorded in stressed (I_3_), non-inoculated plants, even when the latter were sprayed with the higher chitosan concentration (0.50 g.L^− 1^). Overall, biomass allocation increased under I_1_ and in response to chitosan and *Streptomyces*, especially in pods. In contrast, the highest dry weight of pods (32.59 ± 0.46 g) was produced by inoculated plants subjected to a short irrigation interval (8 days) and a low chitosan concentration (0.25 g.L^− 1^) (Fig. 3e). This was followed by the I_1_ treatment with bacterial inoculation and chitosan application at 0.50 g.L^− 1^. Both of these treatments were significantly higher than the I_3_ treatment without bacteria or chitosan. Moreover, the number of pods per plant varied significantly (*p* < 0.05) in response to the different irrigation intervals, seeds inoculation with *Streptomyces* spp., and chitosan concentrations (Fig. 3f). The most substantial effect was observed in the optimally irrigated I_1_ group, where plants that were both inoculated and sprayed with 0.50 g.L^− 1^ chitosan produced the highest number of pods (51.1 ± 1.38). In sharp contrast, the stressed I_3_ group that received neither bacterial inoculation nor chitosan treatment yielded the fewest pods (24.67 ± 1.15). The observed findings indicate a direct positive relationship between water availability and pod set, demonstrating that the onset of water stress at flowering is a critical factor leading to reduced pod numbers. These outcomes were consistent with those of Prince et al. [49], who noted that water stress increased the rate of pod abortion in soybean during the early pod-filling stage, ultimately decreasing the final pod number. The results also suggest that *Streptomyces* inoculation alleviates the adverse effects of water stress by enhancing the accumulation of osmotic regulatory substances, thereby improving soybean drought tolerance. The work of Villafane et al. [48] demonstrate the efficacy of this approach, recording a significantly greater pod number in soybean plants treated with *Streptomyces* N2A + B than in those subjected to the conventional treatment (F + B), which showed the lowest value. Stomatal regulation is also essential for maintaining carbon assimilation under osmotic stress conditions [50]. Accordingly, chitosan has been shown to enhance stomatal conductance, helping plants sustain CO₂ uptake and photosynthetic activity during environmental stress [51]. The resulting boost in photosynthesis may increase carbohydrate production, a finding consistent with the observed improvement in chlorophyll content (Fig. 4a and b). The additional energy provided by these carbohydrates is likely allocated to pod development, a primary sink for photosynthates, ultimately reducing pod abortion and increasing the final number of pods per plant. On the other hand, under water stress conditions (I_3_), the interactive effect of *Streptomyces* inoculation with 0.50 g.L^− 1^ chitosan significantly enhanced plant height, pod number per plant, and pod dry weight relative to the control plants (*p* < 0.05). This indicates that, under water stress, the combined application of *Streptomyces* and chitosan created a synergistic bio-stimulant and bio-protective effect. Such dual priming may have potentiated the physiological responses in soybean plants, leading to enhanced stress tolerance, better maintenance of photosynthesis and turgor, and a more favorable hormonal balance. Consequently, plants allocated more resources to growth and reproduction, resulting in significant improvement of these agronomic traits compared to stressed control plants without this protective synergy. 

### Effects of irrigation intervals, *Streptomyces* inoculation, and chitosan on biochemical traits

According to the ANOVA results (Table 3), irrigation intervals had a highly significant (*p* < 0.001) effect on all physiological and biochemical parameters (i.e., chlorophyll a, chlorophyll b, relative water content (RWC), proline, peroxidase, and polyphenol oxidase activity). Both seed inoculation with *Streptomyces* spp. and chitosan application were also highly significant across most traits (*p* < 0.001), except for polyphenol oxidase activity, for which the effect was significant at *p* < 0.01. Regarding two-way interactions, irrigation × bacterial inoculation notably affected photosynthetic pigments (chlorophyll a and b), proline content, peroxidase activity, and polyphenol oxidase activity, with no significant impact on RWC. The irrigation × chitosan interaction significantly influenced all measured traits, with the most potent effects on proline content and polyphenol oxidase activity (*p* < 0.001). Moreover, the interaction between bacterial inoculation and chitosan was highly significant for RWC, proline content, and polyphenol oxidase activity (*p* < 0.001), and significant for chlorophyll b (*p* < 0.01). However, this interaction had no significant effect on chlorophyll a and peroxidase activity. Finally, the three-way interaction (irrigation intervals × bacterial inoculation × chitosan) was statistically significant for all biochemical traits and antioxidant enzyme activities except RWC (*p* > 0.05).

**Table 3:** Analysis of variance (ANOVA) for the effects of irrigation intervals, *Streptomyces* inoculation, and Chitosan application on biochemical characteristics of soybean

Source of variance	d.f	Chlorophyll a	Chlorophyll b	Relative water content	Proline content	Peroxidase activity	Polyphenol oxidase activity
Blocks	2	0.008 ^ns^	0.012 ^ns^	78.70 ^***^	1.35 ^ns^	0.03 ^***^	0.30 ^**^
I	2	28.899 ^***^	11.71 ^***^	2156.89 ^***^	0.04 ^***^	0.29 ^***^	5.44 ^***^
Error (a)	4	0.010	0.024	0.59	1.65	3.72	0.015
B	1	4.369 ^***^	2.51 ^***^	142.98 ^***^	0.010^***^	0.06 ^***^	0.209 ^**^
I × B	2	0.384 ^***^	0.18 ^*^	0.132 ^ns^	3.42 ^***^	0.01 ^**^	0.270 ^**^
Error (b)	6	0.013	0.02	0.199	1.29	4.27	0.014
C	2	2.132 ^***^	3.13 ^***^	170.00 ^***^	4.02 ^***^	0.010 ^***^	0.750 ^**^
I × C	4	0.154 ^**^	0.21 ^**^	2.211 ^*^	3.77 ^***^	0.002 ^*^	0.281 ^***^
B × C	2	0.028 ^ns^	0.28 ^**^	14.07 ^***^	1.19 ^***^	8.58 ^ns^	0.660 ^***^
I × B × C	4	0.131 ^*^	0.10 ^*^	1.53 ^ns^	1.32 ^***^	0.002 ^**^	0.117 ^***^
Error (c)	24	0.031	0.03	0.70	3.78	6.66	0.001

I = Irrigation intervals, B = *Streptomyces* inoculation, C = Chitosan application

*, **, *** = Significant at *p* < 0.05, *p* < 0.01, and *p* < 0.001, respectively; ns = non-significant

The combined effects of irrigation intervals, seed inoculation, and chitosan application on the biochemical characteristics and antioxidant enzyme activity of soybean are presented in Fig. 4. Chlorophyll content is a key stress indicator, as various abiotic stresses can reduce both pigment concentration and light absorption capacity. Although water stress can initially stimulate chlorophyll synthesis, the associated reduction in the uptake of essential elements ultimately impairs photosynthetic function [52]. This pattern was reflected in our results, where plants irrigated every 8 days had significantly higher chlorophyll a and b levels than water-stressed plants (irrigated every 18 days). The reduction in chlorophyll content under water stress is mainly ascribed to the downregulation of the *cab* gene family, which impairs the synthesis of the major chlorophyll-protein complexes essential for photosynthesis [53]. The results indicated that, averaged across irrigation intervals, the combined application of *Streptomyces* inoculation and chitosan considerably enhanced chlorophyll a and b content (Fig. 4a and b). The interaction between short irrigation interval, seed inoculation with *Streptomyces* and foliar spraying of chitosan at 0.50 g.L^− 1^ resulted in the highest chlorophyll a, and b levels (7.16 ± 0.58 and 4.13 ± 0.59 mg/g FW, respectively). Contrarily, the stressed plants without bacterial inoculation or chitosan treatment showed significantly lower values (3.00 ± 0.01 and 1.07 ± 0.11 mg/g FW, respectively). This reduction was possibly ascribed to the accumulation of reactive oxygen species (ROS) under deficit irrigation conditions, which promote the oxidative degradation of chloroplast structures and weakens chlorophyll biosynthesis [54–56]. The application of chitosan (at 0.50 g.L^− 1^) mitigated this degradation, thereby preserving chlorophyll content, consistent with the previous findings of Khan et al. [57]. This protective effect may stem from chitosan’s ability to modulate chloroplast gene expression, improving chloroplast ultrastructure. This effect is synergistically complemented by PGPR, such as *Streptomyces* spp., which increase chlorophyll content, potentially through modulation of stress hormones like abscisic acid (ABA) under water deficit conditions [58]. Tiwari et al. [59] also established a direct relationship between bacterial inoculation and chlorophyll preservation, showing that by alleviating water stress, the bacteria prevent chlorophyll degradation. This may lead to enhanced photosynthetic activity and subsequent plant growth. Relative water content (RWC) is a vital indicator of water status in plants, reflecting the balance between water supply to the leaf tissue and transpiration rates [60]. A high RWC helps plants mitigate reactive oxygen species and osmotic stresses induced by water deficit, which can contribute to greater yield [61]. In this study, however, the combination of irrigation, *Streptomyces* inoculation, and chitosan application did not produce a significant interactive effect on RWC. This may be because the irrigation intervals created a stress level where the plant’s intrinsic capacity to maintain RWC was already saturated. Under these conditions, the bio-stimulants may have operated through similar, non-complementary pathways to reach that maximum threshold. Alternatively, RWC may simply be a less sensitive indicator of the fine-tuned hormonal or biochemical synergies induced by the combined treatment compared to other measured parameters, reflecting a hierarchy in trait responsiveness to complex bio-stimulant interactions. Conversely, proline content, a key osmolyte and marker for stress tolerance [62], was substantially affected by the three-way interaction of these factors (Fig. 4c). Proline accumulation increased directly with the length of the irrigation interval, reaching its highest level (0.438 ± 0.006 mg/g FW) in the stressed treatment (I_3_) that received both bacterial inoculation and 0.50 g.L^− 1^ chitosan. This value was remarkably (*p* < 0.05) higher than that observed in the well-watered I_1_ treatment without bio-stimulants (0.297 ± 0.009 mg/g FW). Notably, under the I_3_ treatment, proline levels with 0.50 g.L^− 1^ chitosan were statistically similar with or without bacterial inoculation (0.438 ± 0.006 and 0.417 ± 0.004 mg/g FW, respectively). This suggests that under water stress (I_3_), the osmotic stress is the primary driver of proline accumulation. The physiological role of this accumulated proline is to maintain cellular turgor and facilitate water uptake, consistent with its function as a key osmoprotectant that stabilizes proteins and membranes, thereby reducing oxidative damage [63]. Under water deficit stress induced by extended irrigation intervals, soybean plants inoculated with *Streptomyces* spp. exhibited a marked increase in proline content relative to non-inoculated plants. *Streptomyces* does not directly produce proline but acts as a bio-stimulant, enhancing the plant’s innate defense mechanisms and consequently augmenting their resilience to water stress. Shan et al. [24] also demonstrated that inoculation of wheat with *Streptomyces pactum* Act12 significantly increased proline and soluble protein content, boosted antioxidant enzyme activity, and reduced oxidative stress. Moreover, chitosan plays a significant and well-documented role in enhancing plant stress tolerance, partly by promoting the proline accumulation, which is in line with the outcomes of the present study (Fig. 4c). This is linked to its ability to elevate abscisic acid (ABA) levels, a key stress-response hormone that triggers stomatal closure and activates stress-responsive genes, including those involved in proline biosynthesis. The efficacy of this mechanism is supported by studies on white clover and thyme, which also reported higher proline levels following chitosan treatment under water stress conditions [64, 65]. Beyond osmotic adjustment, the antioxidant defense system was also significantly influenced. Statistical analysis revealed that antioxidant enzyme activity varied significantly across the interaction among irrigation, bacterial inoculation, and chitosan application. For instance, peroxidase activity was most strongly induced by the combination of a long irrigation interval (I_3_) with both bacteria and 0.50 g.L^− 1^ chitosan, yielding a maximum value of 0.670 ± 0.10 ∆A₄₂₅/g FW/15 min (Fig. 4d). The opposite conditions, well-watered (I_1_) without bacteria or chitosan, resulted in the minimum peroxidase activity (0.276 ± 0.003 ∆A₄₂₅/g FW/15 min). Furthermore, polyphenol oxidase activity was also significantly affected, showing a 90.99% increase in stressed plants (I_3_) treated with bacteria and 0.50 g.L^− 1^ chitosan compared to those under the I_2_ (13-day) irrigation regime without any treatment (Fig. 4e). These results suggest that the applied treatments, particularly the bio-stimulants, mitigated water stress damage through two complementary mechanisms: improving plant water status via osmotic adjustment and suppressing oxidative stress via elevated antioxidant enzyme activities. The data further indicate that *Streptomyces* inoculation specifically potentiated this antioxidant response, enabling efficient ROS detoxification. This protective effect preserved photosynthetic function and cellular integrity, thereby supporting yield stability, a finding consistent with previous reports on chitosan’s capacity to stimulate antioxidant defenses in other crops [66]. Under water stress, compared to the control, plants treated with both *Streptomyces* and chitosan (particularly at 0.50 g.L^− 1^) exhibited significantly greater values (*p* < 0.05) for key biochemical parameters, including chlorophyll a and b, proline content, and peroxidase and polyphenol oxidase activity. This implies that the synergistic application alleviated drought stress by enhancing critical protective and metabolic processes. Specifically, the significant increase in proline content facilitated osmotic adjustment and cellular protection, while the elevated activities of peroxidase and polyphenol oxidase strengthened the antioxidant system to mitigate oxidative damage. Concurrently, the maintenance of higher chlorophyll levels supported sustained photosynthetic activity. This integrated physiological enhancement enabled plants to maintain growth and reproductive development under drought conditions.

### Effects of irrigation intervals, *Streptomyces* inoculation, and chitosan on yield-related traits

The ANOVA results (Table 4) indicated that the combined effect of irrigation intervals, *Streptomyces* inoculation, and chitosan application significantly influenced soybean yield and its components. The main effects of irrigation, bacterial inoculation, and chitosan application were individually significant for all measured parameters (i.e., number of pods/plants, weight of pods/plant, weight of 100-seed, and seed yield). Moreover, all the two-way interactions notably influenced most traits. The only exceptions were the number of pods per plant and seed yield in the irrigation × bacterial inoculation interaction, which were not significant. Finally, the three-way interaction was highly significant (*p* < 0.001) for number and dry weight of pods and seed yield, and was significant for 100-seed weight (*p* < 0.01). 

**Table 4:** Analysis of variance (ANOVA) for the effects of irrigation intervals, *Streptomyces* inoculation, and Chitosan application on yield and yield components of soybean

Source of variance	d.f	Number of pods per plants	Weight of pods per plant	Weight of 100-seed	Seed yield
Blocks	2	0.29 ^ns^	27.55 ^**^	18.74 ^**^	745212.94 ^***^
I	2	248.13 ^***^	745.20 ^***^	60.20 ^***^	2050070.6 ^***^
Error (a)	4	0.16	0.84	0.87	4173.76
B	1	167.13 ^***^	211.71 ^***^	21.17 ^**^	1208340.2 ^***^
I × B	2	4.57 ^ns^	7.80 ^*^	10.47 ^**^	11426.67 ^ns^
Error (b)	6	3.20	0.97	0.75	2663.09
C	2	71.35 ^***^	97.23 ^***^	17.98 ^***^	364247.9 ^***^
I × C	4	51.96 ^***^	2.38 ^***^	3.70 ^***^	21903.6 ^**^
B × C	2	19.46 ^***^	2.22 ^**^	1.72 ^**^	54302.70 ^***^
I × B × C	4	28.24 ^***^	6.81 ^***^	1.45 ^**^	64140.06 ^***^
Error (c)	24	1.04	0.33	0.23	4640.43

I = Irrigation intervals, B = *Streptomyces* inoculation, C = Chitosan application

*, **, *** = Significant at *p* < 0.05, *p* < 0.01, and *p* < 0.001, respectively; ns = non-significant

**Fig. 5:**
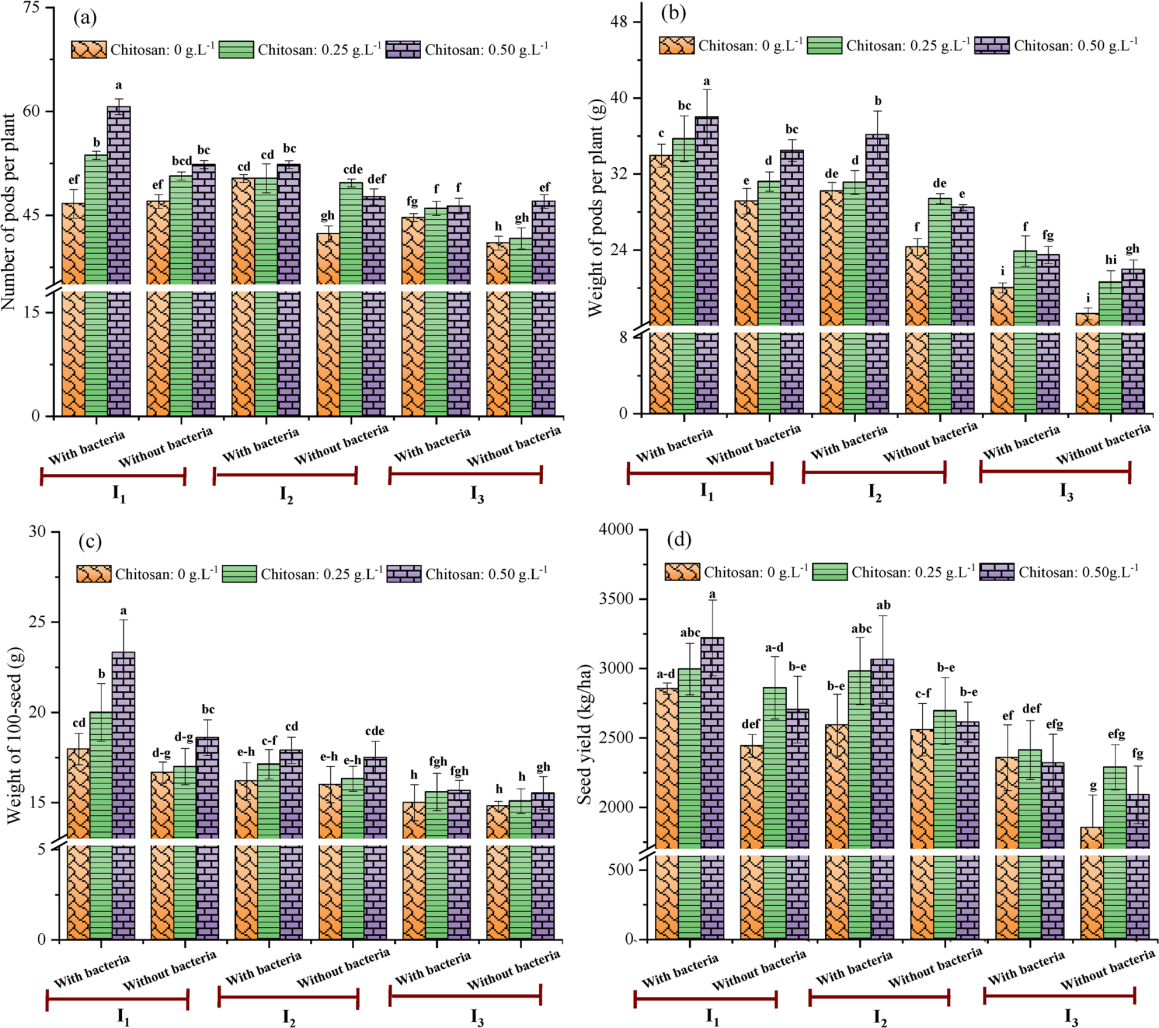
Combined effects of irrigation intervals, *Streptomyces* inoculation, and chitosan on number of pods per plant (**a**), dry weight of pods (**b**), weight of 100-seed (**c**), and seed yield (**d**) of soybean. I_1_ = Irrigation every 8 days, I_2_ = Irrigation every 13 days, I_3_ = Irrigation every 18 days. Different letters on the bars denote statistically significant differences according to Tukey’s test at *p* < 0.05

Figure 5a–d exhibits the effects of irrigation intervals, seed inoculation, and chitosan application on yield and its components of soybean. The number and weight of pods are fundamental and highly reliable indicators of final seed yield. Results show that these traits were strongly influenced by irrigation, as water deficit during the critical flowering and pod-filling stages led to significant decreases. The maximum pod number (60.67 ± 1.15) and the heaviest pods (38.00 ± 2.91 g) were recorded under the well-watered regime (I_1_) with *Streptomyces* inoculation and 0.50 g.L^− 1^ chitosan (Fig. 5a and b). Conversely, the minimum values (41.00 ± 1.00 pods and 17.27 ± 0.62 g) were observed under I_3_ treatment without bio-stimulants. These findings demonstrates that water deficit, particularly during reproductive stages, severely curtails pod set and development. This underscores the critical importance of adequate water availability for soybeans during flowering and pod development stages (R_1_–R_5_). Deficit irrigation during this period accelerates the shift from vegetative to reproductive growth, shortening the flowering and grain-filling phases due to associated high temperatures and moisture stress. This reduces photosynthesis and nutrient assimilation, leading to flower abortion, fewer matured pods, and ultimately lower grain weight and yield [67]. Maintaining optimal soil moisture through short irrigation interval is therefore crucial for minimizing these stresses and enhancing pod retention. Beyond irrigation management, chitosan treatment provides a viable mitigation strategy by enhancing chlorophyll content, thereby boosting photosynthetic capacity and the carbohydrate supply essential for pod and seed development, an effect previously documented in soybeans [68]. Furthermore, the 100-seed weight, a key indicator of soybean yield and seed size, was also significantly influenced by the irrigation treatments (Fig. 5c). The highest 100-seed weight (23.33 ± 0.58 g) was found in well-watered plants (I_1_) combined with bacterial inoculation and chitosan at 0.50 g.L^− 1^, while the lowest (14.81 ± 0.17 g) was achieved under severe deficit irrigation (I_3_) without bio-stimulants, a trend similar to that reported by Karam et al. [69]. This reduction can be attributed to the disruptive effects of water stress during reproductive growth stages on photosynthetic assimilate production, sugar metabolism, and nutrient translocation to the seeds [10]. Remarkably, neither chitosan nor *Streptomyces* inoculation significantly mitigated this reduction under water deficit condition (18-day interval). A potential explanation for this limited efficacy was a physiological trade-off induced by the bio-stimulants. The energetic cost of eliciting defense responses may have diverted metabolic resources away from reproductive processes such as seed filling, instead prioritizing immediate stress tolerance. This concept is supported by the work of Desclaux et al. [12], who found that water stress during seed filling reduced the number of seeds per pod and seed weight.

Moreover, soybean seed yield, a multifactorial trait, is highly vulnerable to drought stress. The results demonstrated that subjecting soybean plants to a long irrigation interval (I_3_) without biostimulant application caused a substantial seed yield reduction of up to 42.45% (Fig. 5d), a decrease consistent with the reductions in pod number, pod weight, and 100-seed weight (Fig. 5a–c). This is in line with reports that soybean yield is highly sensitive to water stress during the reproductive stage, particularly flowering and pod-filling [70]. This period is physiologically critical for determining final yield, as water stress impairs yield architecture by reducing the number of seeds per pod and their individual weight [71]. The resultant yield loss is also attributed to a decrease in photosynthetic carbon assimilation during stress, which directly compromised the resources available for seed development.

Conversely, the highest seed yield (3220.93 ± 273.48 kg/ha) was achieved under frequent irrigation (I_1_) with the combined application of *Streptomyces* and 0.50 g.L^− 1^ chitosan (Fig. 5d). The positive influence of chitosan on seed yield can be explained by its ability to improve the plant’s overall nutritional status by enhancing nutrient uptake and translocation. This effect is linked to chitosan’s role in stimulating physiological activity, promoting vegetative growth, and facilitating the efficient transport of photo-assimilates to developing seeds. These findings were supported by the results of Chibu and Shibayama [68], who observed that chitosan application at early growth stages promoted plant development and increased soybean seed yield. *Streptomyces* inoculation likely contributed to this synergy by improving stress resilience and resource acquisition, leading to the optimal yield observed under the combined treatment. Importantly, under drought (I_3_), the interaction between *Streptomyces* inoculation and chitosan significantly enhanced (*p* < 0.05) seed performance, as evidenced by higher pod number, pod weight, and seed yield per plant compared to the control. This yield advantage can be attributed to a treatment-induced cascade of biochemical and physiological improvements. Specifically, enhanced chlorophyll preservation sustained photosynthesis, while concurrent increases in proline and antioxidant activity protected cellular turgor and integrity. This protection minimized flower and pod abortion, boosting pod set. Subsequently, the sustained photosynthetic source strength and efficient hormonal signaling channeled assimilates into developing seeds, directly enhancing pod weight and final yield.

### Effects of irrigation intervals, *Streptomyces* inoculation, and chitosan on chemical composition of seeds

The analysis of variance exhibited a highly significant main effect (*p* < 0.001) of irrigation, bacterial inoculation, and chitosan on oil content (Table 5). For protein content, the main effect of chitosan was also highly significant (*p* < 0.001). All two-way interactions significantly affected both traits, with the interaction between bacterial inoculation and chitosan having the most potent effect on oil content. Furthermore, the three-way interaction (irrigation × *Streptomyces* × chitosan) was significant for oil (*p* < 0.05) and protein (*p* < 0.01) content. 

### Principal component analysis (PCA)

**Table 5:** Analysis of variance (ANOVA) for the effects of irrigation intervals, *Streptomyces* inoculation, and Chitosan application on oil and protein content of soybean seeds

Source of variance	d.f	Oil content	Protein content
Blocks	2	0.206 ^ns^	0.916 ^ns^
I	2	124.02 ^***^	114.01^**^
Error (a)	4	0.169	1.90
B	1	27.82 ^***^	15.28 ^*^
I × B	2	4.29 ^*^	21.34 ^*^
Error (b)	6	0.529	2.16
C	2	12.82 ^***^	32.43 ^***^
I × C	4	2.086 ^**^	12.18 ^**^
B × C	2	5.90 ^***^	15.63 ^**^
I × B × C	4	1.34 ^*^	10.72 ^**^
Error (c)	24	0.427	2.05

[I = Irrigation intervals, B = *Streptomyces* inoculation, C = Chitosan application

*, **, *** = Significant at *p* < 0.05, *p* < 0.01, and *p* < 0.001, respectively; ns = non-significant

**Fig. 6:**
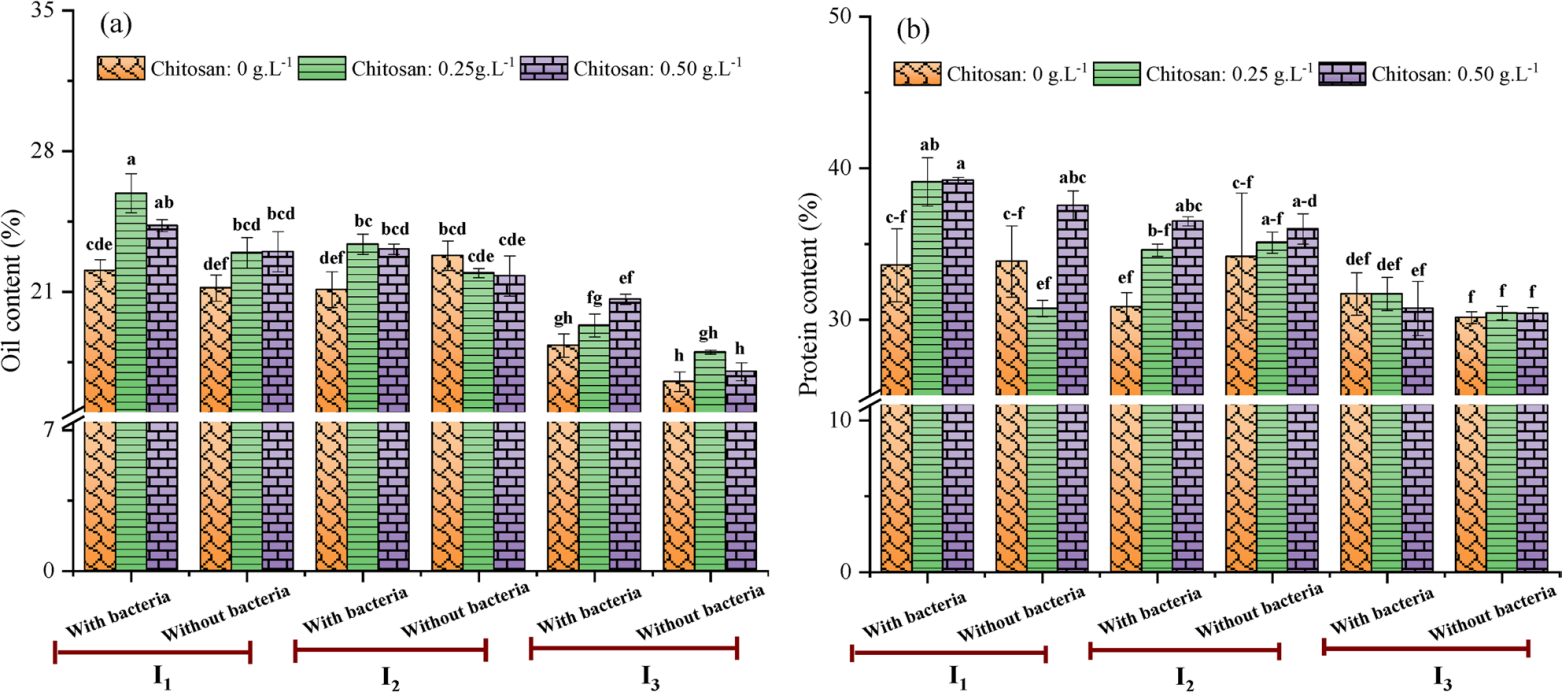
Combined effects of irrigation intervals, *Streptomyces* inoculation, and chitosan on oil content (**a**) and protein content of soybean seeds. I_1_ = Irrigation every 8 days, I_2_ = Irrigation every 13 days, I_3_ = Irrigation every 18 days. Different letters on the bars denote statistically significant differences according to Tukey’s test at *p* < 0.05

Soybean oil is industrially vital as both a premium edible oil and a key renewable feedstock for biodiesel. Biosynthetically, seed oil is derived from photosynthetic carbon fixation in leaves, with subsequent conversion of carbohydrates to triacylglycerols (TAGs) via a compartmentalized metabolic pathway in the endoplasmic reticulum, plastids, and cytosol [72]. The results indicated that oil content varied significantly among treatments (Fig. 6a). Specifically, the interaction between a short irrigation interval (I_1_) and bacterial inoculation, combined with a foliar spray of chitosan at 0.50 g.L^− 1^, maximized oil content (25.91 ± 0.97%). This combination likely enhanced oil biosynthesis by providing the most favorable conditions for metabolic partitioning. The well-watered condition (I_1_) sustained photosynthesis and carbon assimilation, supplying abundant precursors for lipid biosynthesis. Concurrently, the combined bio-stimulants (*S. enissocaesilis* and high-dose chitosan) may have improved phosphate solubilization and phytohormone production while upregulating key enzymes in the lipid synthesis pathway, thereby directing more photo-assimilates toward seed oil production. Contrarily, the minimum value of 16.53% ± 0.49% was recorded under the I_3_ treatment without inoculation or chitosan. Under the well-watered (I_1_) and inoculated conditions, no significant difference in oil content was found between the two chitosan concentrations (0.25 and 0.50 g.L^− 1^). Esmaeeli et al. [11] also reported that water-stressed soybean plants had significantly lower oil content than optimally irrigated plants during the growing season (*p* < 0.05). This reduction was mainly linked to the oxidation of polyunsaturated fatty acids [73] and to an impaired capacity of seeds to absorb photo-assimilates and synthesize oil [74]. 

Protein serves as a crucial energy source for plant embryos, aiding in seedling development [75]. Analysis revealed significant variation in seed protein content, with the maximum value (39.20 ± 0.20%) being achieved through the combined application of the well-watered regime, bacterial inoculation, and 0.50 g.L^− 1^ chitosan (Fig. 6b). This was closely followed by the same irrigation and 0.25 g.L^− 1^ chitosan combined with *Streptomyces* inoculation. The outcome under this combined treatment reflects optimal conditions for nitrogen assimilation and allocation. This suggests that short irrigation interval is critical for nutrient mobility and uptake, particularly nitrate. Simultaneously, the application of bio-stimulants likely enhanced nitrogen availability through biological fixation and improved hormonal balance. This synergy may have shifted the metabolic sink priority in seeds, creating a high nitrogen flux that was efficiently partitioned into storage protein synthesis during grain filling. Conversely, the minimum protein content (30.15 ± 0.39%) was recorded under I_3_ without bacterial inoculation or chitosan application. This finding is consistent with the established understanding that water stress impairs protein biosynthesis. Specifically, it downregulates key storage protein genes, such as *Gy4* and *β-conglycinin*, and reduces photosynthetic rates, thereby depleting the metabolic precursors required for protein synthesis [76, 77]. Furthermore, Ravelombola [78] reported that water stress disrupts protein structure and synthesis pathways, impairs the breakdown of proteins, and thereby hinders amino acids mobilization. However, the relationship between water stress and seed composition is complex, as some studies report conflicting results. For instance, Dornbos and Mullen [79] observed a 4.4% increase in protein content alongside a 2.9% decrease in oil content under water stress. This contrasts with other reports, including the present study and the work of Maleki et al. [80], which confirm a reduction in soybean seed protein under deficit irrigation conditions. Soybean seeds from plants treated with *Streptomyces* or chitosan showed observably higher oil and protein levels under water stress (I_3_) than untreated controls. This improvement is likely due to enhanced plant tolerance to water stress following the application of bio-stimulants (*Streptomyces* and chitosan), which support the metabolic functions essential for oil and protein synthesis. These results demonstrate that both bio-stimulants not only boost crop productivity but also improve the physiological quality of the seeds [29, 48]. Sharifa [81] also observed that the foliar application of chitosan, particularly at 200 ppm, effectively increased protein content relative to unsprayed plants. More recently, Esmaeeli et al. [11] demonstrated that water stress (defined as irrigation after 150 mm of pan evaporation) reduced oil and protein content by an average of 12.5% and 23.3%. Crucially, the application of chitosan at 0.5 g.L^− 1^ mitigated these losses, increasing oil and protein content by 8.1% and 6.7%, respectively, compared to the stressed, untreated control. Interestingly, water-stressed plants treated with the synergistic *Streptomyces*-chitosan application produced significantly greater (*p* < 0.05) oil content than untreated control plants, whereas **a** non-significant increase was observed for protein content. This indicates that the bio-stimulant treatment preferentially channeled photo-assimilates and metabolic resources toward oil biosynthesis over nitrogen assimilation and protein accumulation under drought conditions.

In summary, this study demonstrates that combining a short irrigation interval with bio-stimulants can effectively enhance soybean seed yield and quality, key high-value traits for growers and breeders. This approach provides farmers with a practical method to improve market value, while equipping breeders with the knowledge to select resilient, high-yielding genotypes that maintain quality under varying field conditions. Therefore, these findings offer a bio-based strategy to increase soybean crop value and define target phenotypes for future cultivar development.

### Heatmap analysis

**Fig. 7:**
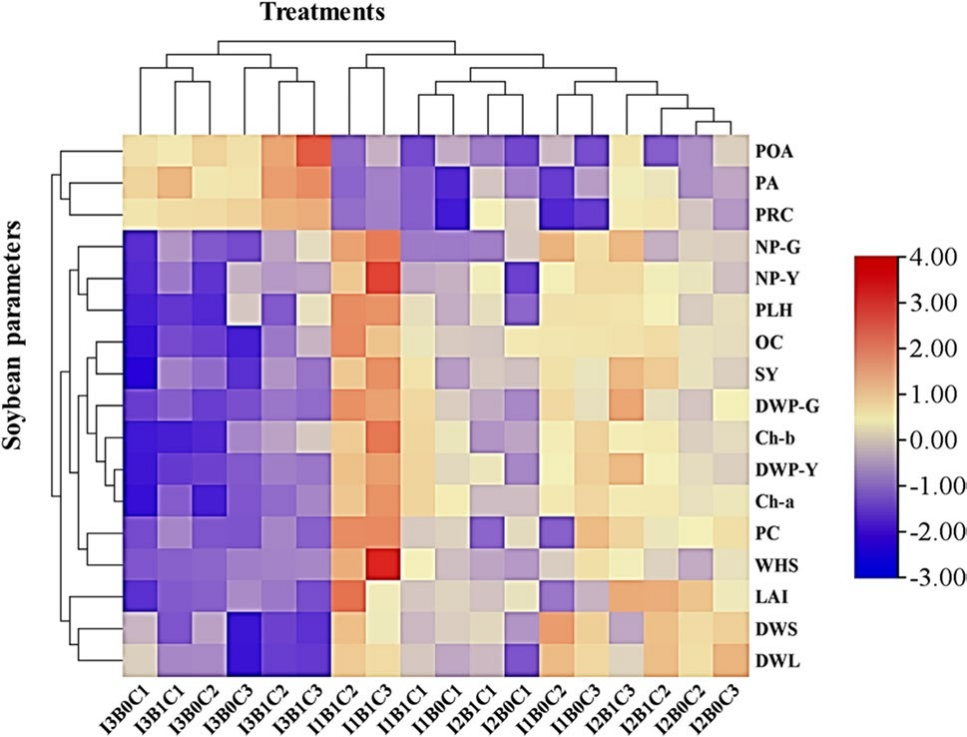
Hierarchical clustering heatmap exhibiting the interactive effects of irrigation intervals, bacterial inoculation, and chitosan application on soybean traits. PHL = plant height (m), LAI = leaf area index, DWL = dry weight of leaves, DWS = dry weight of stem, NP-G = number of pods per plant at growth stage, DWP-G = dry weight of pods at growth stage, Ch-a = chlorophyll a, Ch-b = chlorophyll b, PRC = proline content, PA, peroxidase activity, POA, polyphenol oxidase activity, NP-Y = number of pods per plant at harvest stage, DWP-Y = weight of pods at harvest, WHS = weight of 100-seed, SY = seed yield (t/ha), PC = protein content, OC = oil content. I_1_ = irrigation every 8 days, I_2_ = irrigation every 13 days, I_3_ = irrigation every 18 days, B_0_ = without bacterial inoculation, B_1_ = with bacterial inoculation, C_1_ = 0 g.L^− 1^ chitosan, C_2_ = 0.25 g.L^− 1^ chitosan, C_3_ = 0.50 g.L^− 1^ chitosan

Heatmap analysis effectively visualizes associations between experimental treatments and measured parameters. In this regard, the clustering heatmap was constructed to elucidate the interrelationships between the treatment combinations (irrigation intervals, bacterial inoculation, and chitosan) and the studied traits of soybean, including growth, physiological, yield, and chemical properties (Fig. 7). The heatmap revealed distinct response patterns, grouping the measured characteristics into clear clusters. Plant height (PHL) and pod number at both stages (NP-G, NP-Y) formed one response cluster, showing peak intensity under the short irrigation interval (I_1_) combined with *Streptomyces* inoculation and chitosan. A clear gradient was visible, with PHL intensity peaking at a chitosan concentration of 0.25 g.L^− 1^, while NP-G and NP-Y formed a distinct sub-cluster, reaching their maxima at the higher concentration of 0.50 g.L^− 1^. Furthermore, a coherent cluster comprising photosynthetic pigments (Ch-a, Ch-b) and yield components (DWP-Y, WHS, SY) displayed the most pronounced response, which corresponded exclusively to the combined treatment of short irrigation, *Streptomyces* inoculation, and 0.50 g.L^− 1^ chitosan. This visualization confirms that this specific treatment provided the optimal condition for integrating improved photosynthetic capacity with enhanced partitioning of assimilates to yield. Most notably, despite their separation on the heatmap, DWP-G, LAI, PC, and OC all peaked under the short irrigation treatment combined with inoculation and 0.25 g.L^− 1^ chitosan, revealing a consistent response pattern. DWL and DWS, conversely, showed a strong positive association with moderate irrigation (I_2_) without inoculation but with 0.50 g.L^− 1^ chitosan. This reflects optimized water-use efficiency, in which moderate irrigation, primed by chitosan, enhanced carbon allocation to vegetative structures without competing with the resource demands of microbial symbionts. The I_3_ treatment is visually dominated by cool-toned cells, indicating the lowest values across most measured parameters. This pattern was most pronounced, forming the clearest visual cluster of low responses, in the absence of bacterial inoculation and chitosan. The stress indicator parameters (i.e., PRC, PA, and POA) formed a distinct cluster, with the highest intensities under the long irrigation interval, particularly in treatments combining bacterial inoculation with a higher chitosan concentration. This suggests a potentiated defense response, in which the long irrigation interval served as the primary abiotic stress, and the combination of bacterial inoculation and high-concentration chitosan acted as a powerful biotic elicitor. Overall, hierarchical analysis revealed a clear separation: the I_1_ treatment with inoculation and 0.50 g.L^− 1^ chitosan enhanced most growth and yield-related traits, whereas the identical amendments under the I_3_ interval consistently improved stress indicator parameters.

### Principal component analysis (PCA)

**Fig. 8:**
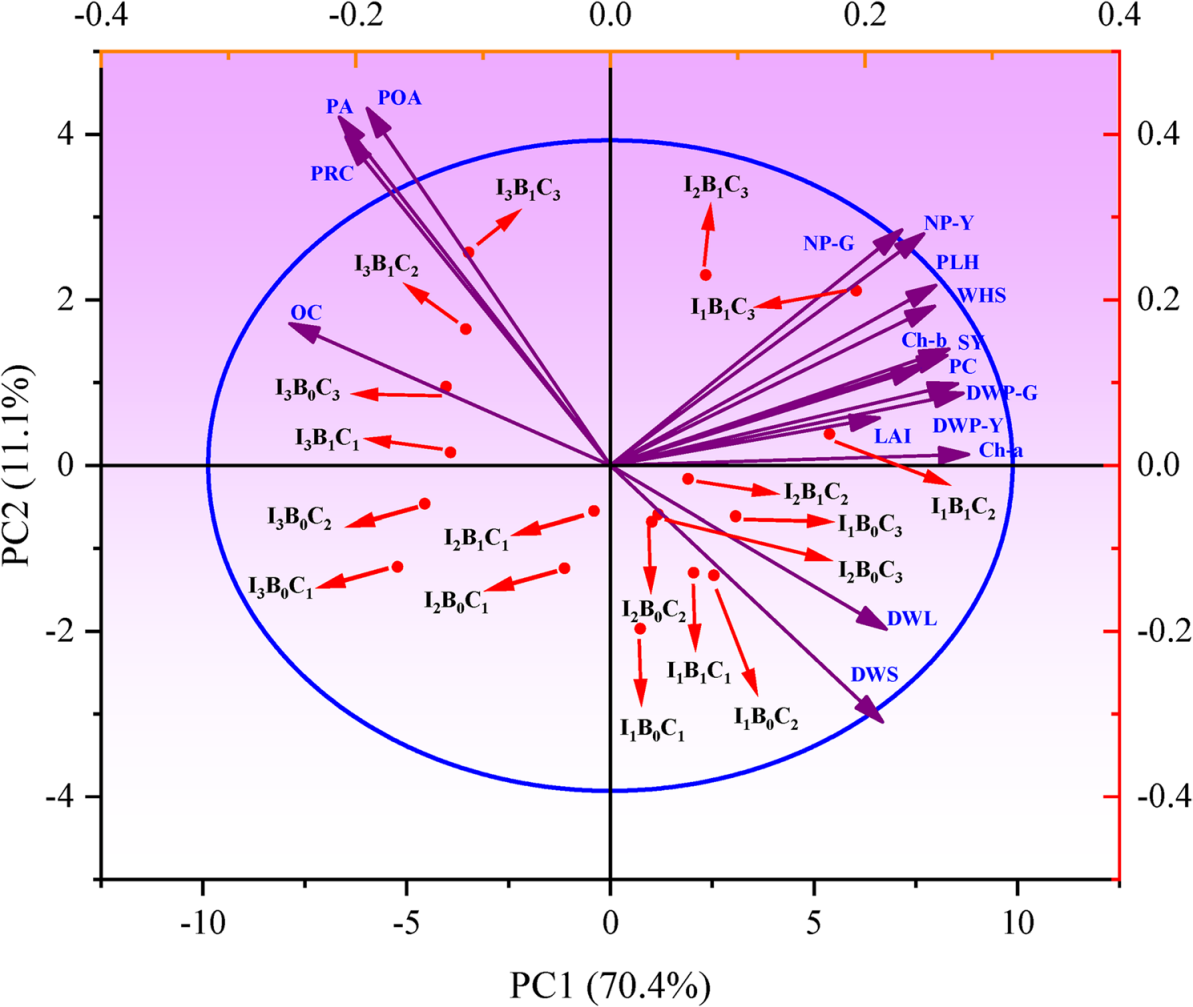
PCA for growth, physiological, biochemical, yield, and chemical properties of soybean under different irrigation intervals, bacterial inoculation, and chitosan application. PHL = plant height, LAI = leaf area index, DWL = dry weight of leaves, DWS = dry weight of stem, NP-G = number of pods per plant at growth stage, DWP-G = dry weight of pods at growth stage, Ch-a = chlorophyll a, Ch-b = chlorophyll b, PRC = proline content, PA, peroxidase activity, POA, polyphenol oxidase activity, NP-Y = number of pods per plant at harvest stage, DWP-Y = weight of pods at harvest, WHS = weight of 100-seed, SY = seed yield, PC = protein content, OC = oil content. I_1_ = irrigation every 8 days, I_2_ = irrigation every 13 days, I_3_ = irrigation every 18 days, B_0_ = without bacterial inoculation, B_1_ = with bacterial inoculation, C_1_ = 0 g.L^− 1^ chitosan, C_2_ = 0.25 g.L^− 1^ chitosan, C_3_ = 0.50 g.L^− 1^ chitosan

PCA was used to simplify the interpretation of combined impacts of irrigation intervals, *Streptomyces* inoculation, and chitosan application on the growth, physiological, biochemical, yield, and chemical characteristics of soybean (Fig. 8). The PCA revealed that 81.50% of the total variance in the measured soybean parameters was explained by the two principal components, with PC1 and PC2 contributing 70.40% and 11.10%, respectively. PC1 was positively associated with high-yield and high-growth traits (e.g., chlorophyll a and b, 100-seed weight, and seed yield), while strongly separating them from negatively loaded stress-response traits (e.g., proline content, peroxidase activity). PC2 (11.10%) primarily differentiated treatments based on traits related to moderate responses and biomass allocation. This cumulative variance is considered sufficient to reliably assess the similarities and variations among treatments [82, 83]. The first cluster, situated in the lower right-hand quadrant with dry weight of leaves and stems (DWL and DWS), comprised treatments that largely lacked *Streptomyces* inoculation (B_0_). This suggests that the absence of microbial symbiosis was a key factor promoting a physiological shift toward increased vegetative biomass production. A distinct, high-performance cluster, located in the positive quadrants of both PC1 and PC2, was formed by treatments combining *Streptomyces* inoculation (B_1_) with moderate-to-high chitosan concentrations under short-to-moderate irrigation (I_1_, I_2_). This synergy promoted an integrated physiological state, which was strongly correlated with a full spectrum of traits ranging from photosynthesis (Ch-a, Ch-b) and vegetative growth (LAI, PHL) to all major yield components (pod number, pod weight, 100-seed weight, and final seed yield). The observed outcomes demonstrate that short and moderate irrigation intervals (I_1_ and I_2_), combined with bacterial inoculation and chitosan application, substantially enhanced soybean growth, photosynthetic efficiency, and yield-related traits. The inoculation and chitosan likely acted as complementary bio-stimulants under favorable soil moisture, synergistically enhancing root function, nutrient uptake, and photosynthetic efficiency. This, in turn, promoted more efficient transport of photo-assimilates from source to sink tissues, ultimately channeling resources toward reproductive growth and integrating improvements across vegetative growth, photosynthesis, and all major yield components. The third cluster grouped the majority of treatments subjected to the long irrigation interval (I_3_), positioning them in the upper left-hand quadrant. This placement indicates that, under significant water stress, the treatments, regardless of inoculation or chitosan concentration, primarily triggered a strong, coordinated biochemical stress response. Such response was characterized by the upregulation of key stress-related compounds and enzymes, including proline content (PRC), peroxidase (PA), and polyphenol oxidase (POA) activity. Conversely, treatments receiving 0 g.L^− 1^ chitosan (C_1_), particularly under moderate to long irrigation intervals (I_2_ and I_3_), clustered independently without strong associations to any specific measured parameters. This implies that chitosan application was necessary to produce a distinct physiological response, irrespective of the irrigation regime or inoculation status.

### Radar plot analysis of treatment effects on soybean traits

**Fig. 9:**
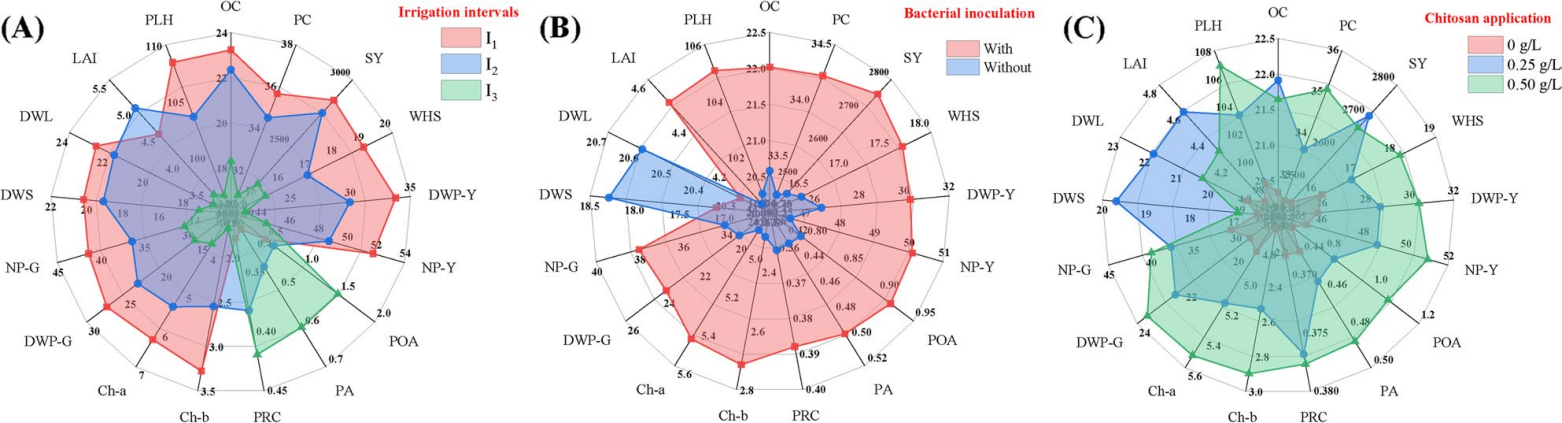
Radar plot illustrating the relative effects of three treatments, irrigation intervals (**A**), bacterial inoculation (**B**), and chitosan application (**C**), on a comprehensive set of soybean traits, including growth, physiological, biochemical, yield, and chemical parameters. Values are scaled from 0% (center) to 100% (outer edge), and a color-coded line denotes the contribution of each treatment. PHL = plant height, LAI = leaf area index, DWL = dry weight of leaves, DWS = dry weight of stem, NP-G = number of pods per plant at growth stage, DWP-G = dry weight of pods at growth stage, Ch-a = chlorophyll a, Ch-b = chlorophyll b, PRC = proline content, PA, peroxidase activity, POA, polyphenol oxidase activity, NP-Y = number of pods per plant at harvest stage, DWP-Y = dry weight of pods at harvest, WHS = weight of 100-seed, SY = seed yield, PC = protein content, OC = oil content

A radar plot (also known as a spider plot) is a multivariate graphical method used here to display the relative value of multiple agronomic parameters for each treatment, with axes radiating from a central point. Radar plot analysis (Fig. 9A–C) revealed a clear hierarchy in the influence of the treatments (i.e., irrigation intervals, bacterial inoculation, and chitosan application) on soybean traits. For comparative visualization, the values for each trait were scaled to a 0–100% range to represent their relative magnitude under each treatment. The I_1_ treatment (red line) had a predominant effect on the majority of photosynthetic pigments, growth, yield, and chemical characteristics, where it accounted for approximately 61–88% of the contribution (Fig. 9A). This was particularly evident for plant height (PLH), oil content (OC), dry weight of pods at harvest (DWP-Y), and chlorophyll-b. I_2_ (blue line) contributed moderately (32–77%) across many traits, with a particularly pronounced effect on oil content (OC) and leaf area (LAI). Conversely, I_3_ (green line) had minimal contributions to most parameters. Still, it was the primary driver of stress-related traits, including proline content and antioxidant enzyme activity (PRC, PA, and POA), accounting for 75–80% of the variation in these traits. Furthermore, Fig. 9B shows that *Streptomyces* inoculation (red line) contributed 66–87% to most growth, physiological, yield, and biochemical parameters, a significantly greater influence than the non-inoculated control (blue line). This effect was most pronounced for PHL, LAI, Ch-a, Ch-b, POA, NP-Y, WHS, and SY. Interestingly, a strong opposite pattern was observed for the dry weight of leaves and stems (DWL, DWS), in which the non-inoculated control (B_0_) was the primary contributor, explaining 75–89% of the variation across these vegetative biomass traits. For the foliar application of chitosan (Fig. 9C), the 0.50 g.L^− 1^ concentration (green line) consistently displayed the highest contribution across most parameters, with values of 70–92%. However, its contribution to DWL, DWS, LAI, OC, and SY was lower than that of the 0.25 g.L^− 1^ treatment. For these specific traits, the 0.25 g.L^− 1^ application (blue line) showed an inverse trend, contributing about 77–92% and thus becoming the dominant factor. The control treatment (0 g.L^− 1^, red line) contributed the least, remaining below 35% for all traits. Collectively, the radar visualization confirmed that the short irrigation interval combined with bacterial inoculation and 0.50 g·L^− 1^ chitosan consistently optimized growth and yield performance, whereas the long irrigation interval was predominantly associated with stress-related parameters.

## Conclusions

Irrigation interval, *Streptomyces* inoculation, and chitosan application significantly affected soybean growth, physiology, yield, and seed composition. The integrated application of seed inoculation and 0.50 g.L^− 1^ chitosan under a short irrigation interval significantly enhanced photosynthetic pigments compared to other treatments. In contrast, the same treatment combination induced the highest antioxidative enzyme activities when applied under a long irrigation interval. Most notably, the well-watered treatment combined with inoculation and 0.50 g.L^− 1^ chitosan produced the highest total seed yield. Conversely, a long irrigation interval substantially reduced oil and protein content, an effect that was most pronounced in the absence of inoculation or chitosan. Critically, these results demonstrate that omitting these bio-stimulants, especially under water stress (I_3_), leads to significant reductions in seed yield and quality, directly impacting market value. Therefore, the synergistic combination of *Streptomyces* inoculation and higher chitosan concentration represents a key strategy for semi-arid farming, optimizing yield when water is available and critically protecting plant function and seed quality during periods of drought stress. Future research should validate this integrated approach under multi-location trials across diverse agro-ecological regions to facilitate its adoption as a standardized, sustainable practice for drought mitigation in legume production. Furthermore, the current lack of a detailed economic analysis underscores the need for subsequent studies to evaluate the profitability and commercial feasibility of these treatments for farmers and stakeholders.

## Data Availability

All data are included in this article, and any further information will be made available from the corresponding author on reasonable request.
